# Survey among retina specialists in Brazil about inflammatory reactions after intravitreal antiangiogenic therapy

**DOI:** 10.5935/0004-2749.20200031

**Published:** 2020

**Authors:** Gustavo Barreto Melo, Magno Ferreira, Maurício Maia

**Affiliations:** 1 Hospital de Olhos de Sergipe, Aracaju, SE, Brazil; 2 Universidade Federal de São Paulo, São Paulo, SP, Brazil; 3 Universidade Federal de Uberlândia, Uberlândia, MG, Brazil

**Keywords:** Retina, Inflammation, Intravitreal injections, Bevacizumab, Ranibizumab, Receptors, vascular endothelial growth factor, Retina, Inflamação, Injeções intravítreas, Bevacizumab, Ranibizumab, Receptores de fatores de crescimento do endotélio vascular

## Abstract

**Purpose:**

This survey aimed at assessing the clinical characteristics of patients with inflammatory reactions after intravitreal injection of antiangiogenic agents and the techniques employed by Brazilian retina specialists.

**Methods:**

We sent an 18-item questionnaire electronically to retina specialists who are using antiangiogenic agents. We got the responses between September 21 and December 23, 2018.

**Results:**

A total of 58 retina specialists participated. Most of them were from Southeastern Brazil (50%), 82.8% were dedicated to both medical and surgical practices, and 86.2% had practiced for more than 5 years. Respondents reported a mean number of 2.14 ± 1.63 patients with inflammation, 44.8% with panuveitis, and 79.3% with onset of symptoms within 72 h. Specialists used aflibercept (53.4%), bevacizumab (29.3%), and ranibizumab (27.6%). Most patients were treated with steroid drops (70.7%), and their inflammation subsided after 11.5 ± 11.5 days (86.2% lacked irreversible complications). The specialists blamed the syringe as the cause of the inflammation in 25.9% of the cases, 41.4% used Becton-Dickinson Ultra-Fine syringes, 43.1% injected the drug at room temperature, and 37.9% removed the air (53.4% by flicking the syringe). Most specialists did not detect silicone oil (67.2%), but 17.2% of them performed vitrectomies to remove vitreous opacities. Finally, 44.8% of specialists injected the same antiangiogenic agent in an eye with prior inflammatory reaction without further inflammation.

**Conclusions:**

Most specialists reported cases of early-onset inflammation after intravitreal injection of antiangiogenic agents. The incidence of irreversible complications was low. Aflibercept was the most common agent used. The causes of inflammation remain unknown, but we formulated some relevant hypotheses.

## Abstract (pt)

**Objetivo:**

Esta pesquisa teve como objetivo avaliar as características clínicas de pacientes com reações inflamatórias após injeção intravítrea de agentes antiangiogênicos e as técnicas empregadas por especialistas em retina brasileiros.

**Métodos:**

Enviamos eletronicamente um questionário de 18 itens para especialistas em retina que usam agentes antiangiogênicos. Recebemos as respostas entre 21 de setembro e 23 de dezembro de 2018.

**Resultados:**

Um total de 58 especialistas em retina participaram. A maioria era do Sudeste do Brasil (50%), 82,8% eram dedicados a práticas médicas e cirúrgicas e 86,2% praticavam há mais de 5 anos. Os entrevistados informaram um número médio de 2,14 ± 1,63 pacientes com inflamação, 44,8% com panuveíte e 79,3% com início dos sintomas dentro de 72 horas. Especialistas utilizaram aflibercepte (53,4%), bevacizumabe (29,3%) e ranibizumabe (26=7,6%). A maioria dos pacientes foi tratada com colírios de esteroides (70,7%), e sua inflamação diminuiu após 11,5 ± 11,5 dias (86,2% não apresentaram complicações irreversíveis). Os especialistas responsabilizaram a seringa como causa da inflamação em 25,9% dos casos, 41,4% usaram seringas Becton-Dickinson Ultra-Fine, 43,1% injetaram a droga em temperatura ambiente e 37,9% removeram o ar (53,4% sacudindo a seringa). A maioria dos especialistas não detectou óleo de silicone (67,2%), mas 17,2% realizaram vitrectomias para remoção de opacidades vítreas. Finalmente, 44,8% dos especialistas injetaram o mesmo agente angiogênicos em um olho com reação inflamatória prévia, sem surgimento de nova inflamação.

**Conclusões:**

A maioria dos especialistas relatou casos de inflamação de início precoce após injeção intravítrea de agentes antiangiogênicos. A incidência complicações irreversíveis foi baixa. Aflibercepte foi o agente mais frequentemente usado. As causas da inflamação permanecem desconhecidas, embora formulamos algumas hipóteses relevantes.

## INTRODUCTION

Inflammation after intravitreal injection of an antiangiogenic drug is an uncommon but worrisome clinical finding that may easily be mistaken for infectious endophthalmitis. Williams et al.^(1)^ reported 0.10% noninfectious vitritis rates after 66,356 bevacizumab injections, 0.02% after 26,161 ranibizumab injections, and 0.16% after 8,071 aflibercept injections. Interestingly, the authors noticed that many cases tended to cluster instead of occurring at a consistent rate each year.

The American Society of Retina Specialists Therapeutic Surveillance Committee received notifications of 56 cases of aflibercept-related sterile inflammation from December 1, 2011, to February 12, 2014^(2)^. However, they did not identify risk factors, and the pathogenesis remained unclear. Another publication showed an association between six cases of inflammation after aflibercept injections with a particular brand of syringe^(3)^, and the authors suspected that the inflammation was caused by silicone oil droplets in the vitreous of the patients. In another study, that same syringe was shown to relea se silicone oil, especially after the agitation caused by flicking it^(4)^.

Considering the scarce knowledge on the possible factors associated with inflammation after antiangiogenic intravitreal injections, studies addressing this issue are warranted. Therefore, we designed this survey for Brazilian retina specialists to assess their techniques and the clinical characteristics of patients with inflammatory reactions after intravitreal antiangiogenic drug injections.

## METHODS

We sent an electronic 18-item questionnaire to 954 members of the Brazilian Retina and Vitreous Society, seeking responses from those who had patients with inflammation after an intravitreal injection. We asked the specialists to exclude any cases of suspected infectious endophthalmitis. The Institutional Review Board of the Federal University of São Paulo approved this study, which followed the tenets of the Declaration of Helsinki.

We obtained responses between September 21, 2018, and December 23, 2018. The survey encompassed questions related to the demographic data of the retina specialists, clinical features and prognoses of their patients, and the techniques adopted while handling the drugs and the syringes at the time of the injection. We encouraged participants to identify themselves for further contact to clarify any doubts related to the questionnaire. We obtained no patient names.

## RESULTS

A total of 58 retina specialists answered the survey. Most of them were men (77.6%), 50% were from Southeastern Brazil, 82.8% were dedicated to both medical and surgical retina practices, and 86.2% had practiced for more than 5 years. They reported a mean number of 2.14 ± 1.63 patients with inflammation (Table 1).

**Table 1:** Demographic information about the retina specialists

	N (%)
Gender	
Male	45 (77.6)
Female	13 (22.4)
Region of practice	N (%)
North	3 (5.2)
Northeast	16 (27.6)
Midwest	6(10.3)
Southeast	29 (50.0)
South	4 (6.9)
Specialty	N (%)
Medical retina	10(17.2)
Surgical and medical retina	48 (82.8)
Time in practice	N (%)
Less than 5 years	8(13.8)
More than 5 years	50 (86.2)
Number of patients with inflammation	Days
Mean ± SD	2.14 ± 1.63
Median	2
Mode	1

SD= standard deviation.

Simultaneous anterior and posterior uveitis were reported by 44.8% of the physicians (Table 2). The onset of symptoms occurred within 72 h of the injections in 79.3% of cases. The specialists used aflibercept (53.4%), bevacizumab (29.3%), and ranibizumab (27.6%) as the drugs in association with inflammation. Most patients were treated with steroid drops (70.7%) and achieved a final resolution after an average of 11.5 ± 11.5 days, and 86.2% remained free from irreversible complications.

**Table 2:** Clinical picture of cases with inflammation

	N (%)
Type of inflammation	
Anterior uveitis	15(25.9)
Vitritis	17(29.3)
Anterior uveitis + vitritis	26 (44.8)
Time to onset	N (%)
Within 24 h	12 (20.7)
Within 48 h	22 (37.9)
Within 72 h	12 (20.7)
Between 4 and 7 days	8(13.8)
Over 7 days	4 (6.9)
Management	N (%)
Observation	5 (8.6)
Topical steroids	41 (70.7)
Topical antibiotics	17(29.3)
Aqueous or vitreous tap	5 (8.6)
Intravitreal antibiotics	11 (19.0)
Vitrectomy	14(24.1)
Drug	N (%)
Aflibercept	31 (53.4)
Bevacizumab	17(29.3)
Ranibizumab	16(27.6)
Time to resolution	Days
Mean ± SD	11.5 ± 11.5
Median	7
Mode	7
Irreversible complication	N (%)
Yes	8(13.8)
No	50 (86.2)

SD= standard deviation.

Table 3 shows that the syringe and problems with the drug were considered the causative factors by 25.9% and 22.4% of specialists, respectively, and 24.1% of them were unaware of possible causes. The specialists used Becton-Dickinson (BD) Ultra-Fine syringes in 41.4% of cases, 43.1% of them injected the drug at room temperature, 37.9% removed any air from the syringe before injecting, 44.8% noticed some air in the eye after the procedure, and 53.4% flicked the syringe to reduce air in the drug solution prior to injecting the agent.

**Table 3:** Technique and associated features

	N (%)
Causes of inflammation	
Unknown	14(24.1)
Inadequate drug temperature	7(12.1)
Inadequate drug maintenance	3 (5.2)
Inadequate drug manufacturing	13 (22.4)
Drug itself	9(15.5)
Syringe	15 (25.9)
Inadequate technique	3 (5.2)
Other reasons	7(12.1)
Syringe model	N (%)
BD Tuberculin/Plastipak	19 (32.8)
BD UltraFine	24 (41.4)
BD SafetyGlide	5 (8.6)
Braun Omnifix-F	2 (3.4)
Descarpack	2 (3.4)
Injex	2 (3.4)
SR	2 (3.4)
Terumo	3 (5.2)
Other	2 (3.4)
1 don’t remember	4 (6.9)
Techniques followed	N (%)
Injecting the cold drug into the eye	22 (37.9)
Injecting the room temperature drug into the eye	25 (43.1)
Avoiding air to enter the syringe when aspirating the drug	4 (6.9)
Allowing air to enter the syringe but removing it completely before injecting	22 (37.9)
Allowing air to enter the syringe but dissociating it from the fluid to avoid eye entry	19 (32.8)
Usually noticing some air in the patient’s eye	26 (44.8)
Actions to avoid air entry into the eye	N (%)
Flicking the syringe until the air is far from the needle side	31 (53.4)
None. Injecting without worrying about the air	5 (8.6)
Gently swinging the syringe until most of the air is far from the needle side	11 (19.0)
Making centrifugal movements until the air is far from the needle side	11 (19.0)
Did any of the patients with inflammation present silicone oil in the eye?	N (%)
Yes. Some	7(12.1)
Yes. All of them	6(10.3)
No	39 (67.2)
Never paid attention to that	6(10.3)
Did any patients undergo vitrectomy to treat floaters after resolution of inflammation?	N (%)
Yes, due to vitreous opacities	10(17.2)
Yes, due to silicone oil droplets	0(0)
No	48 (82.8)
Were those patients with inflammation ever treated with antiangiogenic drugs again?	N (%)
Yes. With the same drug and without inflammation	26 (44.8)
Yes. With a new drug and without inflammation	21 (36.2)
Yes. With the same drug and inflammation recurred	2 (3.4)
No	9(15.5)

BD= Becton-Dickinson; SR= Saldanha-Rodrigues.

Most (67.2%) retina specialists did not detect silicone oil in the eyes, and 17.2% of patients reportedly underwent vitrectomy to remove residual vitreous opacities. Afterward, 44.8% of the physicians injected the same drug without further inflammation (Table 3).

## DISCUSSION

A total of 58 out of 954 retina specialists answered the survey. This number is important because of the published rarity of the inflammatory events (0.02%-0.37% of injections)^(1,5-7)^. The demographic data of the respondents were similar to the overall demographics in the membership database of the Brazilian Retina and Vitreous Society.

The clinical presentations of the cases reported in this survey were similar to those published: quick onset, decline in visual acuity, vitritis, improvement with steroid drops alone, almost complete recovery to the baseline cli nical picture, and low recurrence rate after subsequent injections with the same agent^(1-3,6)^.

Although aflibercept was the most common drug associated to the cases with inflammation (53.4%), the rates for bevacizumab and ranibizumab were not negligible (29.3% and 27.6%, respectively), in agreement with published rates^(1,7)^.

The early bevacizumab cases in the literature may be due to the contaminants from compounding or the manufacturing process (e.g., bacterial endotoxins have been associated to the same lot numbers^(7)^). Williams et al.^(1)^ showed aflibercept cases linked to a single technician preparing the medication. This may be consistent with another study in which one physician was associated with 85% of the cases^(5)^. Therefore, researchers questioned whether the etiology was associated to the handling and preparation of the drug rather than with its manufacturing.

Majority of the retina specialists in this survey attributed the inflammation in their patients to a cause other than the injected drug itself or its manufacturing process (25.9% of them thought the syringe had caused the inflammation). Indeed, another publication from our group also showed the syringe being implicated in the development of inflammation in a cluster of six cases after aflibercept injection^(3)^, and it associated the silicone oil droplets released after agitation by flicking to the complication. Interestingly, 53.4% of the physicians responding this survey flicked the syringe before injecting. The possible association between inflammation with syringes and silicone oils debated at academic meetings may have influenced the respondents’ opinions.

Becton-Dickinson Ultra-Fine syringes with staked-in needles were the most frequently used models (41.4% of the respondents), and these syringes have been shown to release significant amounts of silicone oil droplets in a light microscopy analysis^(8)^.

Whether the drug temperature impacts the development of inflammation is unclear. In this survey, the numbers of specialists injecting under different room temperatures were similar. Most specialists made an effort to avoid the entry of air into the eye when handling the syringe (especially by flicking), but 44.8% saw air in the vitreous of their patients.

Some studies have associated silicone oil-water interfaces (siliconized syringe walls), air-water interfaces (air bubbles), and agitation stress (flicking of the syringe) with protein aggregation and particle formation^(9-11)^. The highest particle concentrations were found in agitated, siliconized syringes containing air bubbles^(10)^. These studies suggest that flicking the syringe to minimize the presence of air in the vitreous cavity promotes particle formation. Additionally, silicone oil droplets have been reported to act as immunologic adjuvants^(12)^. Finally, another study confirmed the undesirable effects of silicone oil on therapeutic proteins, including adsorption to sili cone oil droplets and increased secretion of innate cytokines from the human peripheral blood mononuclear cells of a small donor panel^(13)^. These results indicate that the oil droplets form complexes with pharmaceutical proteins that may invoke earlyand late-stage immune responses. Therefore, we suspect that the inflammation after intravitreal antiangiogenic injection may be caused by those interactions.

Contradicting our thoughts, most physicians (67.2%) failed to detect silicone oil in the eye of their patients. However, this finding should be interpreted with caution, since majority of silicone oil droplets are tiny and difficult to be seen if not carefully sought. Some studies have reported rates of silicone oil in the vitreous as low as 0.03%-1.7%^(14,15)^. However, a study from our group found it to be as high as 76% in a consecutive series of patients undergoing routine intravitreal injections (unpublished data).^(16)^

In conclusion, retina specialists in Brazil reported that most cases of inflammation after intravitreal antiangiogenic therapy developed early and had a low rate of irreversible complications. Aflibercept was more frequently implicated. The causes of inflammation remain unknown, but we formulated some hypotheses.
